# Clinicians’ attitudes and perceptions of healthcare approaches to environmental sustainability within clinical nutrition: An international survey

**DOI:** 10.1016/j.intf.2026.100369

**Published:** 2026-04-22

**Authors:** A. Bond, D. Cardenas, S. Lal

**Affiliations:** a Intestinal Failure Unit, Salford Royal Foundation Trust, Salford, United Kingdom; b University of Manchester, School of Medicine, Manchester, United Kingdom; c Nutrition Unit, Institut Gustave Roussy, Villejuif, France

**Keywords:** Sustainability, Clinical nutrition, Perceptions, Barriers

## Abstract

**Introduction:**

As global awareness of environmental challenges grows, integrating sustainability into clinical nutrition practices is essential to support both human and planetary health. Within healthcare systems internationally there are a number of barriers to enhanced sustainability which need to be understood, in order for them to be overcome.

**Method:**

A bespoke questionnaire-based survey was developed and disseminated internationally to healthcare professionals working in the sphere of clinical nutrition. Quantitative and qualitative analysis was performed on the recorded responses. Included within the survey was the Sustainability Attitudes Scale (SAS).

**Results:**

A total of 126 responses were collated from 18 countries worldwide. Dietitians comprised the largest represented profession with 35%, followed by doctors 32%. There was a strong opinion that sustainability in a very important aspect of respondents considerations, both in and outside of their working lives. 99% stated they would be in support of changes aimed at making their clinical practice less harmful to the environment. 87% stated that they had never received any formal training in improving sustainability, with a lack of knowledge or experience being recognized as a significant barrier to sustainability focused working practices. 4% stated that it would not be acceptable to consider bearing the potential higher financial cost.

**Conclusion:**

The findings highlight a clear enthusiasm among clinical nutrition professionals for integrating sustainability into practice, despite widespread gaps in formal training and knowledge. Addressing these educational and systemic barriers is crucial to empowering healthcare providers to adopt environmentally responsible approaches.

## Introduction

Environmental sustainability within healthcare systems is rapidly emerging as a critical focus area, yet its integration into clinical nutrition remains underexplored [1]. Indeed, clinical nutrition, which plays a vital role in patient care and the promotion of long-term health outcomes, intersects with sustainability in numerous ways, such as through the sourcing, production and distribution of nutritional products, as well as in the management of food waste [1]. Successful changes in this area of healthcare to mitigate the effect on the environment include efforts to reduce food waste in hospitals through improved inventory management and portion control, and initiatives to prioritize the use of locally sourced, seasonal ingredients in meal preparation to minimize environmental impact. Additionally, advancements in packaging technology for nutritional products have led to more eco-friendly alternatives that decrease reliance on single-use plastics, showcasing a shift toward ‘greener’ practices within the field [2], [3], [4].

However, barriers to broader adoption of sustainable approaches persist in many areas of healthcare delivery. These include a lack of awareness or education among healthcare professionals about the environmental implications of clinical practices and limited infrastructure to implement such changes effectively [5]. Economic constraints further complicate the transition, as sustainable options are often perceived as more costly [6]. Moreover, organizational inertia and resistance to change can hinder the adoption of innovative solutions, while cultural and regional differences may influence perceptions of sustainability and the feasibility of certain interventions.

The integration of sustainability within clinical nutrition carries significant ethical implications, emphasizing the obligation of healthcare professionals to act in the best interests of both patients and the planet [7], [8], [9]. Ethical considerations include the responsibility to minimize environmental harm while ensuring equitable access to nutritional care that meets diverse patient needs. For instance, sourcing sustainable ingredients must balance ecological benefits with cultural sensitivity and affordability, ensuring that no patient group is disadvantaged. To uphold these ethical principles, healthcare professionals require robust education and literacy in sustainability.

Thus, as the global healthcare community strives to mitigate environmental impacts and transition toward sustainable environmental practices, understanding the attitudes and perceptions of healthcare professionals is key to identifying barriers, opportunities, and actionable strategies within this context [10], [11], [12], [13]. However, to-date, the attitudes of clinicians working in the field of clinical nutrition towards environmentally-sustainable practices have not been explored.

## Method

The aim of the study was to conduct a survey amongst clinical nutrition clinicians on their attitudes towards sustainable health care delivery. In the absence of a previous study in this area, a systematic approach was employed to design the survey. Initially, clear objectives were established to define the survey's focus, including assessing awareness, attitudes, or perceived barriers. A literature review informed question design, leveraging insight from prior research on sustainability in healthcare. Questions were appropriately tailored towards the field of clinical nutrition and crafted using a variety of formats, including Likert scales, multiple-choice options, and open-ended prompts, in order to capture both quantitative and qualitative data. Pilot testing was conducted with a small cohort of healthcare professionals to ensure clarity, reliability, and validity of the survey items. Based on feedback, refinements were made to optimize question phrasing and survey structure.

Included within the survey was the Sustainability Attitudes Scale (SAS), a tool designed to measure individual’s attitudes toward sustainability across environmental, social and economic domains. Developed by researchers at Central College and Michigan State University, the SAS consists of 11 statements rated on a six-point scale, ranging from "strongly disagree" to "strongly agree." It is primarily used to assess attitudes in adults, particularly in educational and program evaluation contexts [14]. Remaining questions are detailed within the full survey in Appendix 1.

Respondents were invited to complete the survey electronically, either via a dedicated hyperlink or QR code. Completion was anonymous, with distribution being undertaken through open channels such as social media, news items within national nutrition societies or being made available at national and international education events. Since patient-level data were not collected, ethics approval was not required for this study. Qualitative analysis was performed using a publicly available online Large Language Model (Microsoft Co-pilot).

## Results

**Fig. 1:**
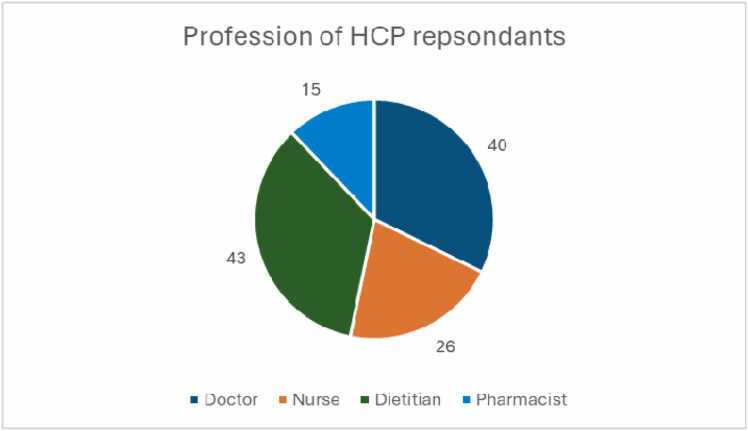
Pie chart demonstrating the number of each type of Health Care Professional responding to survey.

A total of 126 responses were collated from 18 countries worldwide, (Australia, Belgium, Canada, Denmark, Estonia, Ireland, Israel, Italy, Netherlands, New Zealand, Poland, Solvenia, Spain, Sri Lanka, Sweden, Switzerland, UAE, UK) with the UK accounting for 72% of all responses. The mean age of those responding was 41 years (range 23–71), with female respondents accounting for 73%. Dietitians comprised the largest represented profession with 35%, followed by doctors 32% (Fig. 1).

## Attitudes & experience of sustainable practices: sustainability attitudes scale

**Fig. 2:**
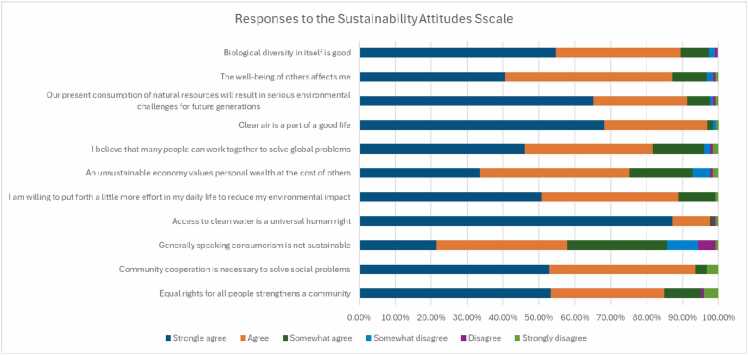
Bar chart demonstrating the participant responses to the SAS.

Responses to the Sustainability Attitudes Scale (SAS) are shown in Fig. 2. In general responses indicated strong agreement on social equality, cooperation and environmental responsibility. A significant proportion of respondents believed that equal rights strengthen communities (53.2% strongly agreed) and that collective efforts can solve global challenges (46% strongly agreed). Clean water was seen as a universal right, with a majority (87.3%) in strong agreement. Views on consumerism being sustainable received a more mixed response, with 27.8% somewhat agreeing and 8.7% somewhat disagreeing. Most respondents recognized the negative impact of excessive resource use on future generations, with 65.1% in strong agreement. Willingness to reduce environmental impact was high (50.8% strongly agree), and clean air was widely valued (68.3% strongly agreed). Overall, the data suggest a prevailing consensus on social and environmental concerns, with minor variations in perspectives on economic sustainability and consumer habits.

When asked to describe the importance of personal (outside of work) and professional (at work) responsibility to promote environmentally friendly and sustainable practice, 96.1% and 82.6% agreed, respectively. Indeed, 95/126 (75.3%) stated that it was extremely important for health care systems (i.e. hospital, homecare services, pharmaceutical companies, other) to work in a way that supports environmentally-sustainable practices. Despite this, only 38% (49/126) respondents felt that the healthcare system they worked in actively supported sustainable practice. However, 125/126 (99%) stated they would be in support of changes aimed at making their clinical practice less harmful to the environment. Notably, only 4% (5/126) stated that it would not be acceptable to consider accepting the potential higher financial cost of more sustainable practice within their healthcare system.

## Education & training in sustainable healthcare delivery

**Fig. 3:**
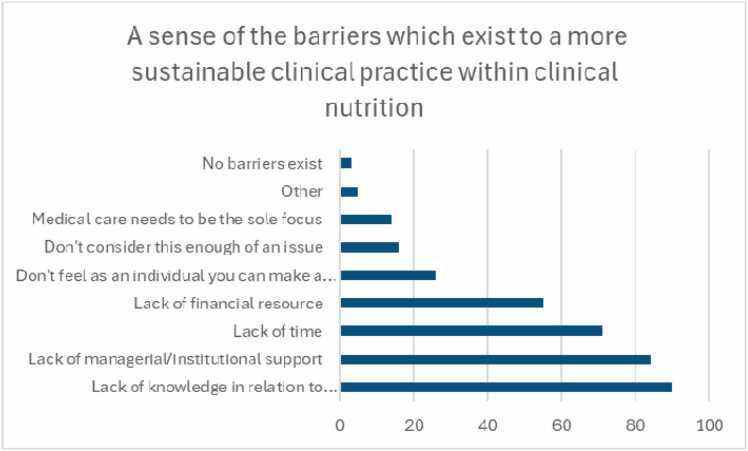
Bar chart demonstrating the number of responses for perceived barriers to implementation of improved sustainability measures into clinical nutrition care.

One hundred and ten respondents (87%) stated that they had never received any formal training in improving sustainability and helping the environment within their workplace, whilst only 5 respondents (4%) stated they had received training in carbon literacy and footprint assessment. Furthermore, only 8 (6%) respondents had previously actively attempted to measure the carbon emissions associated with their delivery of clinical care. When asked how likely respondents were to want to undergo dedicated training in the area of sustainability (1 =not at all, 10 =definitely), there was a mean response of 7.74, with 75/136 responding with 8 or above. Notably, 90/126 (71.4%) of respondents highlighted lack of knowledge as a barrier to implementing change to improve sustainability into their everyday clinical practice. This was the most commonly reported barrier, followed by lack of institutional support and lack of time (Fig. 3).

## Sustainability within clinical nutrition

When perceptions of the environmental impact of clinical nutrition care delivery were assessed (1 = not impactful at all, 10 = extremely impactful), the average rating was 7.17, with 43% rating it 8 or above. When explored further with qualitative analysis, commonly report themes included:

• **Significant concerns about waste and lack of recycling**: A dominant theme was the high level of waste associated with nutritional care, particularly plastic waste from PN bags, feed bottles and other single-use items. Respondents frequently mentioned the lack of, or inadequate, recycling facilities for these materials.
• **Impact of home parenteral nutrition (HPN)**: HPN was specifically highlighted as having a significant environmental impact due to the volume of supplies and associated waste.
• **Inefficiencies in logistics and supply chain**: Issues around transport costs, delivery efficiency, and the potential for wasted supplies were raised.
• **Focus on immediate patient needs**: Some respondents acknowledged the importance of sustainability but prioritized immediate clinical needs and patient care, suggesting a potential tension between these priorities.
• **Lack of knowledge and awareness**: A number of respondents admitted to being unsure about the environmental impact of their practices, indicating a need for better data and information.
• **Desire for more sustainable practices and awareness**: There was a general recognition of the importance of sustainability and a desire to improve practices. Many respondents felt they would benefit from more training and education in this area.
• **Barriers to implementing sustainable practices**: Several barriers were identified, including lack of infrastructure, time constraints due to staffing shortages, cost considerations and a perceived lack of organizational priority.
• **Need for systemic change and leadership**: Many respondents believed that individual efforts are not enough and called for systematic changes, embedded practices and enforcement from higher levels within their healthcare systems.

**Fig. 4:**
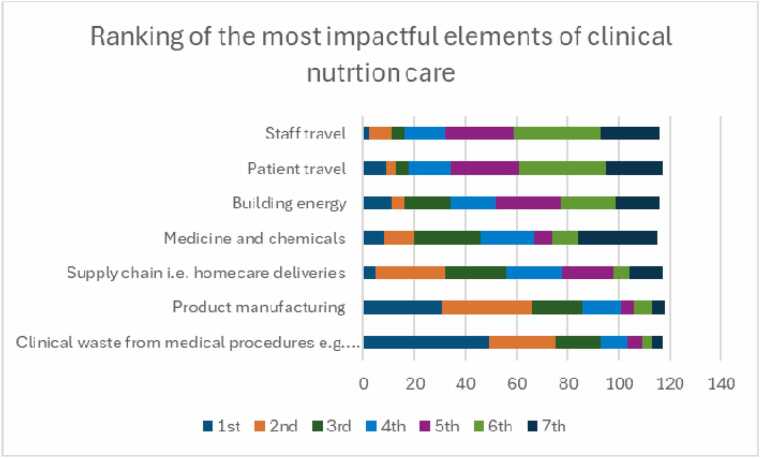
Participant ranking perception of the clinical nutrition care delivery factors which are most environmentally impactful. 1 =most and 7 =least.

When asked to rank the components of clinical nutrition care in terms of their adverse impact on the environment. (1 =most, 7 = least), clinical waste and product manufacturing were felt to be the most impactful, with patient and staff travel were perceived as the least impactful (Fig. 4).

**Fig. 5:**
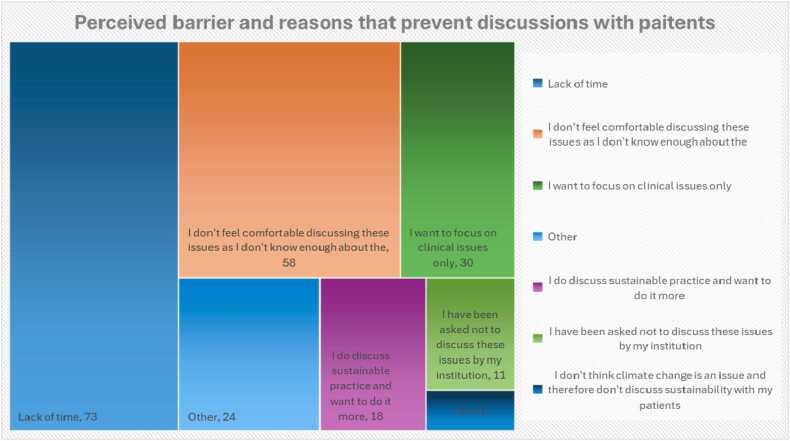
Hierarchy chart reporting the perceived barriers that prevent patient discussions.

When asked about the likelihood of discussing sustainability and environmental issues with their patients, of the clinicians surveyed, 9/126 stated they would actively aim to discuss this, with the remaining 117 stating they were passive to the issue or actively avoided it. A lack of time and subject matter knowledge were the main reasons clinicians did not discuss sustainability and environmentally friendly practices with their patients (Fig. 5).

## Qualitative analysis of narrative responses received

Respondents were asked to explain their response to the question related to what degree their healthcare institution supports sustainable practice. Overarching themes identified included:

• **Lack of systematic or widespread sustainability practices:** A significant portion of respondents indicated that sustainability was not a major priority or consistently implemented across their healthcare system.
• **Recycling is a key concern and area for improvement:** Many comments specifically mentioned the lack of or inadequate recycling facilities and practices as a major issue.
• **Single-Use plastics are a significant problem:** The overuse and difficulty in reducing single-use plastics in the healthcare setting was a recurring concern.
• **Awareness vs. Action:** Respondents noted that, while there might be some awareness or discussion about sustainability, it did not always translate into concrete action or changes in practice by their institution.
• **Individual efforts vs. organizational strategy:** Some respondents described individual efforts and passion for sustainability but pointed to a lack of overall organizational strategy or directive.
• **Cost as a barrier:** Several responses suggested that cost was often prioritized over sustainability when making decisions.
• **Waste management issues:** General waste management, including food waste, medical waste and packaging waste, was identified as a problem area.
• **Potential for improvement and desire for change:** Despite the reservations outlined, many respondents expressed a desire for improvement and suggested specific areas where changes could be made.

Also described by respondents were perceived barriers to sustainable clinical nutrition care delivery; the most frequently described barriers were:

• **Lack of organizational priority:** with little strategic focus.
• **Cost:** being prioritized over sustainability.
• **Inertia/Resistance to change:** amongst clinicians to changes.
• **Logistical challenges:** for example, difficulty controlling plastic waste in a hospital setting.
• **Staff compliance:** low staff compliance with existing initiatives, such as????.
• **Focus on infection prevention:** It was noted that infection prevention sometimes takes precedence over sustainability.

## Discussion

This is the first study to evaluate healthcare professionals’ attitudes to environmentally-sustainable practices withing the field of clinical nutrition. While there was a clear will and desire amongst respondents to undertake clinical nutrition care in a more sustainable manner, the vast majority of clinicians reported a lack of knowledge and skills required to implement environmentally-sustainable endeavors into their daily practice. Overcoming healthcare professionals' lack of knowledge in carbon literacy and sustainable clinical practice requires a multifaceted approach that integrates education, policy changes, and institutional support. One effective strategy would be to incorporate sustainability into medical and healthcare curricula [15]. For example, The Carbon Literacy Project has developed specialized training programs for healthcare workers to enhance their understanding of climate change and its implications for patient care. Specialty and undergraduate medical education is changing to incorporate sustainability training [15], [16]. Additionally, healthcare institutions can implement continuous professional development programs that provide learning opportunities, such as online modules or workshops tailored to different specialties. Governmental and clinical societies would need to adopt new policies in order to implement and adopt these dedicated educational programs.

Leadership support is also crucial—hospital administrators and policymakers should prioritize sustainability initiatives by embedding carbon reduction strategies into clinical guidelines and operational policies. Research has shown that nurses and other healthcare professionals are more likely to engage in sustainable practices when they receive institutional backing and clear guidance [17]. Clinicians surveyed suggest that a lack of managerial support was a frequently encountered barrier. Although we did not receive responses from hospital administrators, it is possible that this lack of support reflects a lack of knowledge or skills amongst managerial teams, again demonstrating the need for additional education amongst all professions involved in healthcare delivery. Furthermore, fostering a culture of sustainability within healthcare settings can encourage professionals to adopt environmentally responsible behaviors in their personal lives. This can be achieved through peer-led initiatives, sustainability champions within departments, and interdisciplinary collaboration to share best practices. By addressing knowledge gaps through education, policy reinforcement, and cultural shifts, healthcare professionals can become active contributors to reducing the carbon footprint of clinical care. An example of how specialist societies can be effective in this area can be seen within the British Association of Parenteral and Enteral Nutrition specialist interest group for sustainable healthcare delivery, which has played a pivotal role by championing education, sharing best practice on a national level and acting in a advocacy capacity, working in collaboration with national patient groups [18].

Lack of institutional engagement, inertia, a lack of time and financial resource where further common barriers reported by respondents. Institutional inertia presents a significant barrier to sustainability efforts in healthcare, as entrenched systems resist change due to bureaucratic complexity, financial constraints, and cultural norms [19]. Healthcare organizations often struggle to implement sustainable practices due to rigid administrative structures and a lack of long-term investment in green initiatives. Leadership hesitancy and inadequate staff training further hinder progress, making it difficult to integrate environmentally-friendly policies into daily operations [20]. Additionally, external pressures such as regulatory requirements and short-term financial goals discourage institutions from prioritizing sustainability. Addressing institutional inertia requires policy reforms, financial incentives and a cultural shift toward sustainability in healthcare management. The extra financial cost, referred to as the Green Premium [1], can often be prohibitive to new initiatives and technologies. This may reduce as technologies advance and production becomes cheaper but, it the interim, reliance upon governmental tax incentive or subsidies may be the best way to bridge the gap.

A significant contributor to the environmental impact of nutritional care delivery identified by the survey was the generation of clinical and plastic waste. The routine use of single-use plastics — such as enteral feeding tubes, packaging for nutritional supplements, and disposable delivery systems and personal protection (e.g. latex gloves) — results in considerable waste volumes that may require specialized disposal. This matter is further complicated by the amount of ancillaries required to provide nutritional support at a patient’s homes, meaning clinical waste is managed through domestic wates provisions. This not only increases the carbon footprint of nutritional care, but also poses challenges for waste management at home, as well as within healthcare institutions and beyond. Addressing the environmental consequences of such waste calls for a re-evaluation of material usage, improved segregation and recycling pathways, as well as innovation in the development of more sustainable alternatives, all while upholding stringent infection control standards [21], [22], [23].

We acknowledge that there are inherent limitations when adopting this type of survey approach. Namely that limited or restricted distribution can introduce bias dependent upon where and how the survey is distributed. We attempted to overcome this by using a number of different processes of distribution. We specifically wanted to target professionals working within clinical nutrition and intestinal failure.

To address the perceived barrier to delivering economically-efficient clinical care, sustainability in healthcare must be balanced with maintaining high-quality patient care and ensuring equitable access. Implementing green initiatives, such as energy-efficient infrastructure and waste reduction, can lower environmental impact without compromising treatment quality [24]. Healthcare systems must prioritize sustainable procurement, reduce carbon emissions, and integrate eco-friendly practices while safeguarding affordability and accessibility. Underscoring these changes, as always, rests on cultural shifts and an emphasis on education from the undergraduate to the postgraduate level, with constant reminders through continued professional development programs. Only then can we ensure that healthcare professionals have the necessary tools to uphold the ethical principles of equity and justice, take responsibility for minimizing environmental impact, and act in the best interests of the patient.

## Patient or guardian consent

There was no patient or guardian consent required as part of the study.

## Funding

There is no funding to declare as part of the study.

## ethical statement

Relevant approval was sort for the study.

## CRediT authorship contribution statement

**Simon Lal:** Writing – review & editing, Writing – original draft, Methodology, Conceptualization. **Cardenas-Braz Diana:** Writing – review & editing, Conceptualization. **Bond Ashley:** Writing – review & editing, Writing – original draft, Methodology, Investigation, Formal analysis, Data curation, Conceptualization.

## Declaration of Competing Interest

The authors declare that they have no known competing financial interests or personal relationships that could have appeared to influence the work reported in this paper.

## Footnotes

Appendix A Supplementary data associated with this article can be found in the online version at doi:10.1016/j.intf.2026.100369.
