# Supplementary material for: Clinicians’ attitudes and perceptions of healthcare approaches to environmental sustainability within clinical nutrition: An international survey
Source: Intest Fail. 2026 Apr 22;10:100369. doi: 10.1016/j.intf.2026.100369 (PMC13112476; doi:10.1016/j.intf.2026.100369)
Supplement: Supplementary file 1 — Supplementary material [file mmc1.pdf]

Health Care Professionals Attitudes to Sustainability within Clinical Nutrition

Thank you for taking the time to complete this short survey.

1. Please select the option which best describes you

- ◯ Male
- ◯ Female
- ◯ Non-Binary
- ◯ Prefer not to say

2. How old are you? (years)

3. In which country do you live?

4. Which option best describes your clinical role?

- ◯ Doctor (Medical)
- ◯ Doctor (Surgeon)
- ◯ Nurse
- ◯ Dietitian
- ◯ Pharmacist

5. To what extent do you agree that individual behaviour to support the environment and sustainable practices is important?

	Strongly agree	Agree	Neutral	Disagree	Strongly disagree
At work	◯	◯	◯	◯	◯
Outside of work	◯	◯	◯	◯	◯

6. How important do you think it is for the health care system (i.e. hospital, homecare services, pharmaceutical companies, other) to work in a way that supports sustainable practices?

- ◯ Extremely important
- ◯ Somewhat important
- ◯ Neutral
- ◯ Somewhat unimportant
- ◯ Extremely unimportant

7. To what extent do you agree that the healthcare system (e.g. hospital, homecare service, other) you work within actively supports sustainable practices?

- ◯ Strongly agree
- ◯ Agree
- ◯ Neutral
- ◯ Disagree
- ◯ Strongly disagree
- ◯ I don't know

8. Could you explain your response to question 7 in a short description?

9. Have you ever had any formal training in improving sustainability and helping the environment within your workplace?

- Yes
- No

10. Have you ever had any formal training at your workplace in relation to carbon literacy?

- Yes
- No
- Never heard of the term until this survey

11. Were it to be offered, how likely would you be to want to undergo dedicated training in the area of sustainability? (1= not at all, 10=definitely)

12. Have you ever measured the carbon footprint of elements of the clinical care that you provide?

- Yes
- No
- No, as I don't know how to measure

13. Please select from the list below your sense of the barriers which exist to a more sustainable clinical practice within clinical nutrition. You may select as many options as you perceive to be true.

- Lack of time
- Lack of managerial/institutional support
- No barriers exist
- Lack of financial resource
- Lack of knowledge in relation to implementing the required change
- Don't consider this enough of an issue
- Don't feel as an individual you can make a significant enough difference
- Medical care needs to be the sole focus
- Other

14. Please rate how you feel about the environmental impact of the clinical nutrition care you provide. (1= not impactful at all, 10= extremely impactful)

☐ ☐ ☐ ☐ ☐ ☐ ☐ ☐ ☐ ☐

15. Could you explain why you choose your response to question 13 below.

[ ]

16. How likely are you to support changes that would be aimed at making your clinical practice less harmful to the environment?

- ○ Very likely
- ○ Somewhat likely
- ○ Neither likely nor unlikely
- ○ Somewhat unlikely
- ○ Very unlikely

17. As part of your current practice how likely are you to discuss sustainable practices and the environment with your patients?

0	1	2	3	4	5	6	7	8	9	10

Not at all likely — Extremely likely

18. Please select the barriers or reasons that you perceive that prevent you from discussing sustainable practices with your patients. Choose as many options as you feel appropriate

- ☐ Lack of time
- ☐ I don't think climate change is an issue and therefore don't discuss sustainability with my patients
- ☐ I want to focus on clinical issues only
- ☐ I do discuss sustainable practice and want to do it more
- ☐ I have been asked not to discuss these issues by my institution
- ☐ I don't feel comfortable discussing these issues as I don't know enough about the topic
- ☐ Other

19. Would you consider the potential higher financial costs related to sustainable practices as one worth accepting in order to reduce harm to the environment?

- ◯ Yes
- ◯ No
- ◯ Maybe

20. Please rank the following components of clinical nutrition care in terms of their adverse impact on the environment. (1=most, 7= least)

- Patient travel
- Staff travel
- Supply chain i.e. homecare deliveries
- Product manufacturing
- Clinical waste from medical procedures e.g. enteral feeding plastics and parenteral support administration
- Building energy
- Medicine and chemicals
- Other - please list
- I don't know

21. Please use the scale below to respond to each statement. This is the Sustainability Attitudes Scale as defined in the introduction statement.

	Strongly disagree	Disagree	Somewhat disagree	Somewhat agree	Agree	Strongly Agree
Equal rights for all people strengthens a community	○	○	○	○	○	○
Community cooperation is necessary to solve social problems	○	○	○	○	○	○
Generally speaking consumerism is not sustainable	○	○	○	○	○	○
Access to clean water is a universal human right	○	○	○	○	○	○
I am willing to put forth a little more effort in my daily life to reduce my environmental impact	○	○	○	○	○	○
An unsustainable economy values personal wealth at the cost of others	○	○	○	○	○	○
I believe that many people can work together to solve global problems	○	○	○	○	○	○
Clear air is a part of a good life	○	○	○	○	○	○
Our present consumption of natural resources will result in serious environmental challenges for future generations	○	○	○	○	○	○
The well-being of others affects me	○	○	○	○	○	○
Biological diversity in itself is good	○	○	○	○	○	○

This content is neither created nor endorsed by Microsoft. The data you submit will be sent to the form owner.

Microsoft Forms
